# The New Gravity: How Generative AI Remapped Innovation and What Academia Must Do Next

**DOI:** 10.2196/89741

**Published:** 2026-10-19

**Authors:** Hossein Akbarialiabad, Ayman Grada, Sancy A Leachman, David Rhew, Alireza Haghighi

**Affiliations:** 1 Department of Dermatology, Oregon Health & Science University, Portland, OR, United States; 2 Faculty of Medicine and Health, University of New South Wales (UNSW Sydney), Sydney, NSW, Australia; 3 Department of Dermatology, Case Western Reserve University School of Medicine, Cleveland, OH, United States; 4 Healthcare, Microsoft, Redmond, WA, United States; 5 Harvard Medical School, 77 Avenue Louis Pasteur, Boston, MA, 02115, United States; 6 Broad Institute of MIT and Harvard, Cambridge, MA, United States; 7 Department of Medicine, Brigham and Women’s Hospital, Harvard Medical School, Boston, MA, United States

**Keywords:** education, innovation, AI, generative AI, academic medical centers, industry

## Abstract

Artificial intelligence is rapidly reshaping clinical research, diagnostics, health system operations, and medical education. Frontier generative AI development is increasingly concentrated in private industry, whose computational infrastructure, proprietary datasets, and capital exceed those of most academic institutions. Although not designed exclusively for medicine, these systems increasingly influence clinical applications. This shift raises a question for academic medical centers (AMCs): What role remains when leading AI systems emerge outside universities? This viewpoint examines industry’s growing role in generative AI, clarifies AMCs’ responsibilities, and proposes a framework for AI governance and literacy across undergraduate, graduate, and continuing medical education (CME). Rather than compete with industry on computational scale, AMCs should reinforce functions for which AMCs have distinctive strengths. These include (1) mechanistic inquiry linking algorithmic outputs to biological and pathophysiologic understanding; (2) interdisciplinary synthesis integrating clinical, ethical, legal, and sociotechnical perspectives; (3) independent and methodologically rigorous validation of clinical AI tools; and (4) institutional stewardship of ethical governance for patient populations that are often underrepresented in commercial datasets. As nonprofit institutions training clinicians, AMCs have a distinct role in safeguarding transparency, fairness, and accountability in AI-enabled health care. We position this 4-role framework to refine AI governance and translational medicine approaches, clarifying which responsibilities AMCs should retain while collaborating with industry. The approach includes longitudinal AI literacy training across undergraduate medical education, graduate medical education, and CME, standardized pathways for evaluating clinical AI, stronger conflict-of-interest safeguards, and complementary partnerships with industry and regulators. We also outline educational assessment methods and safety-monitoring processes, including competency-based assessment across undergraduate medical education, graduate medical education, and CME, postdeployment monitoring, and clear escalation triggers. Embedding verification habits and governance into training and practice can help ensure that generative AI supports clinical reasoning while preserving trust, equity, and scientific rigor. This viewpoint addresses academic leaders, curriculum committees, residency directors, faculty developers, and accreditors. Safe clinical AI requires sustained academic leadership.

## The Changing Landscape of AI Innovation

In 2023, industry produced 51 notable machine learning models, compared with 15 from academia; industry-academia collaborations produced a further 21 models [1]. Although these models are not necessarily designed for medicine, this imbalance reflects where computational power, proprietary data, and capital for frontier development are now concentrated [2]. The term “frontier” in this context refers to general-purpose foundation models trained at scale with broad, cross-domain capabilities rather than task-specific clinical algorithms [3]. Their architectural innovations increasingly shape downstream clinical tools through fine-tuning, API integration, and embedded workflow systems [4]. This concentration has only deepened since then. By 2024, close to 9 in 10 notable AI models originated from industry, and by 2025, the share exceeded 90%, even as academia remained the leading institutional source of highly cited AI research [5,6]. The split is therefore not simply that industry builds more; it is that the 2 sectors are pulling apart along different axes, with industry concentrating on scale and academia concentrating on the kind of scrutiny that scale does not buy.

As a result, the concentration of frontier development in private laboratories indirectly shapes the technical substrate upon which many clinical AI applications are built [7]. For academic medical centers (AMCs), this does not mean disengagement from innovation, but rather a reorientation toward evaluating and governing the clinical instantiations of these broader technological advances [8].

At the same time, the higher education landscape is undergoing its own structural strain. Data from the Federal Reserve Bank of New York document underemployment among recent US college graduates working in jobs that typically do not require a college degree [9]. While not specific to medicine, this trend reflects a broader misalignment between traditional degree pathways and rapidly evolving labor-market expectations in technology-intensive fields [10-12]. For medical education, the implication is not job displacement, but curricular relevance: training programs must ensure that physicians graduate with competencies that remain durable in environments increasingly shaped by algorithmic systems [11,13]. Accreditation bodies increasingly emphasize competency-based education; AI literacy must become part of that competency architecture [14].

Together, these forces pose a profound challenge for AMCs [15]. As the locus of innovation shifts toward industry and the educational pipeline struggles to keep pace with technological change, AMCs may risk marginalization if institutional adaptation lags behind technological transformation [10].

For AMCs, the priority is not to match the computational scale or speed of industry, but to focus on the scientific, educational, and public service functions for which they are structurally best suited (Table 1) [16]. For example, the 2024 AI Index estimated training compute costs of US $78 million for GPT-4 and US $191 million for Gemini Ultra [17]. Analyses suggest that a substantial proportion of newly trained AI PhDs now enter private-sector laboratories [18]. Academic institutions care for diverse patient populations, including Medicaid beneficiaries, Medicare recipients with complex conditions, and newly arrived immigrants. AMCs should evaluate whether clinical AI performs equitably across the populations they serve [19,20]. These asymmetries underscore the need for independent academic oversight to prevent AI deployment from exacerbating existing health inequities.

Effective oversight of clinical AI will require close coordination between academic investigators and national regulatory bodies [21,22]. Academic institutions can generate independent evidence, characterize model limitations, and inform federal standards. Regulators retain statutory enforcement responsibilities, while AMCs remain responsible for institutional oversight of procurement and clinical use [23]. Many industry actors have also strengthened internal validation and compliance infrastructures; however, independent academic evaluation remains essential to mitigate structural conflicts of interest and to preserve public credibility [24,25].

The central question, therefore, is how academic medicine can fulfill its mission when the most powerful AI systems are developed outside university walls. The answer does not lie in replicating industry scale, but in strengthening roles for which AMCs have distinctive strengths: mechanistic inquiry, transparent validation, ethical stewardship, and workforce training. Industry-academia research collaborations can support knowledge cocreation, although differences in priorities and time horizons require explicit management [26]. It is important to recognize that industry is not a monolith; it includes multinational technology corporations, venture-backed startups, nonprofit research laboratories, and public-private partnerships, each operating under distinct governance structures and accountability mechanisms. These developments are constructive and should be welcomed. However, structural differences remain: commercial entities are ultimately accountable to investors and product timelines, whereas academic institutions operate under nonprofit missions centered on patient welfare, education, and public accountability. These differing incentive structures underscore why independent academic evaluation and governance remain essential complements to industry innovation rather than redundant efforts.

This viewpoint seeks to (1) examine the structural shift in generative AI development toward private industry; (2) articulate the distinct and enduring roles of AMCs in ensuring scientific rigor, ethical governance, and educational integrity; and (3) outline a concrete implementation framework for integrating AI literacy and oversight across undergraduate medical education (UME), graduate medical education (GME), and continuing professional development. The intended audience includes academic medical leaders, curriculum designers, residency program directors, faculty development committees, and accreditation stakeholders navigating the integration of AI into clinical training.

**Table 1.** The 4 distinctive roles of academic medical centers (AMCs) in clinical AI.

Role	Core function	What to remember (one-liner)	Evaluation anchor/example
1. Foundational mechanistic inquiry	Wet-lab and translational studies that determine whether AI-suggested biomarkers or therapeutic targets reflect true pathophysiologic mechanisms	AMCs explain *why* models work or fail biologically	Experimental validation of AI-generated hypotheses through bench and translational research
2. Interdisciplinary synthesis	Integrates clinical medicine, biomedicine, computer and social sciences, ethics, and law	Evaluates not only what AI *can* do but what it *should* do and when	Multifaculty AI governance councils within AMCs
3. Independent validation and assurance	Serves as an impartial evaluator of bias, reproducibility, and generalizability in clinical AI tools	Provides staged safety and performance assessment across the AI life cycle	Clinical AI validation pipelines modeled on drug trial frameworks
4. Institutional governance and trust stewardship	Defines procurement standards, privacy safeguards, and equity frameworks	Preserves the public’s trust in responsible AI care	Dedicated AI safety and governance boards with authority analogous to IRBs^a^ or P&T^b^ committees, responsible for auditing model performance, procurement decisions, and clinical integration

a IRB: institutional review board.

b P&T: pharmacy and therapeutics.

## Engaging Credible Counterpositions

Several considerations qualify a simple division between academia and industry. Industry-academia collaborations can support knowledge cocreation [26]. The examples below illustrate ways to combine academic expertise with industry resources. Such arrangements should be assessed on their methods, governance, and safeguards for independent evaluation.

These developments illustrate that the landscape is neither static nor binary. However, even in collaborative or hybrid models, independent academic oversight remains structurally important to preserve methodological neutrality, mitigate conflicts of interest, and maintain public trust. In other words, the question is not whether AMCs should collaborate, but where they must retain independence to evaluate and govern.

Several collaborations illustrate how academic evaluation and industry-scale deployment can be combined without collapsing independence. In radiology, an academic AI development and evaluation laboratory has paired with a large clinical network to translate academic validation methods into life cycle monitoring tools meant for use well beyond a single site, combining research-grade evaluation with deployment experience across thousands of clinical locations [27]. A national specialty society, working with a commercial data partner, has launched an assessment laboratory that evaluates cardiovascular risk algorithms against safety and efficacy standards before health systems adopt them [28]. The Dana-Farber Cancer Institute implemented GPT4DFCI for operations, administration, and research, with direct clinical care explicitly excluded [29]. Further, precompetitive research consortia in the mold of the MIT-IBM Watson AI Lab have pooled academic and corporate resources for shared, publishable work [30]. These arrangements are productive, and the argument here is not that academic medicine should stand apart from them. Academic partners can strengthen these collaborations by providing evaluation protected from sponsor influence through transparent funding, conflict-of-interest safeguards, and the right to publish unfavorable findings.

## Steps Forward: Four Academic Functions That Do Not Require Frontier-Scale Compute

Before turning to the 4 roles, it is worth being precise about why these functions resist substitution, since the claim that they are distinctively academic has to be argued rather than asserted. Industry and hybrid bodies have not been idle on governance. The Coalition for Health AI, a multistakeholder group spanning health systems, technology companies, patient advocates, and academic centers, has issued consensus best-practice guides for trustworthy AI, and its work with the Joint Commission produced sector guidance on responsible use [31]. Specialty societies have begun to establish assessment laboratories of their own, and most large vendors now maintain internal validation and audit functions. These efforts have value; the question is whether they can substitute for institutionally independent oversight. The trajectory of the Coalition’s flagship proposal is instructive. Fierce Healthcare reported that its proposed national network of AI assurance laboratories was discontinued as the Coalition shifted its focus toward AI governance and postdeployment monitoring [32]. The design and sustainability of assurance arrangements require attention alongside evaluation methods. Institutional independence is central to credible evaluation, but it is not guaranteed by nonprofit status. AMCs must protect independence through transparent funding arrangements, conflict-of-interest controls, access to data, and the right to publish unfavorable findings. Commercial organizations may face investor expectations and product deadlines, whereas AMCs also carry obligations to patients, trainees, and the public. AMCs should preserve the ability to publish null or unfavorable findings without sponsor interference.

The 4-role framework that follows is best understood as an extension of an existing model rather than a wholly new one. The translational science spectrum, conventionally described as the T0 through T4 phases that carry a discovery from basic mechanism to population-level effectiveness, already gives medicine a vocabulary for staged, rigorous evaluation, and recent work has begun to map AI and machine learning development onto it, placing exploratory model work at the early stages and clinical-grade tools at T2 and beyond [33]. The roles proposed here track that spectrum: mechanistic inquiry corresponds to the early, explanatory end; independent validation to the clinical testing phases; and governance and trust stewardship to the implementation and population phases, with interdisciplinary synthesis running across all of them as a connective function. This framework also sits alongside, rather than in competition with, the process-oriented governance frameworks now in circulation: the National Institute of Standards and Technology (NIST) AI Risk Management Framework, the World Health Organization’s guidance on AI for health, the Coalition for Health AI blueprints, and the various health care AI governance maturity models. Those frameworks specify what activities a responsible actor should perform; what they largely leave open is which actor is structurally suited to perform which activity. The contribution of the 4-role framework is to make that actor-specific argument explicit for academic medicine: it recasts the translational spectrum not as a research pipeline but as a distribution of institutional responsibilities, and identifies the subset that cannot be delegated without losing the independence that gives it meaning.

First, AMCs must champion foundational mechanistic inquiry, addressing a limitation of many predictive AI systems: associations do not, by themselves, establish causal mechanisms [34]. Although industry focuses on commercially viable applications, academia can explore foundational questions: when an AI model predicts disease progression, does it reflect true biological pathways or statistical artifacts [35]? Understanding why a prediction works is crucial for advancing beyond pattern recognition to genuine medical insight and determining when a tool can be safely generalized. This translational bridge between computational prediction and biological mechanism remains a domain where academic laboratories possess deep methodological expertise.

The importance of mechanistic grounding is particularly evident in areas such as oncology, radiology, and predictive analytics for chronic disease [36]. An algorithm may identify imaging patterns associated with malignancy or electronic health record variables predictive of sepsis; however, without understanding whether these signals correspond to underlying biological pathways, causal drivers, or merely correlational artifacts embedded in the training data, clinical generalization becomes precarious [37]. History offers cautionary examples of models that performed well in development cohorts but failed when underlying workflows or demographic distributions shifted. Academic environments are uniquely positioned to interrogate these discrepancies through prospective trials, translational validation studies, and integration with laboratory-based investigation. By linking model outputs to mechanistic hypotheses and testing them across diverse populations, AMCs can move clinical AI from statistical association to scientifically interpretable insight [35].

Fulfilling this responsibility represents a distinctive contribution to academic leadership. Commercial developers can also fund mechanistic work, although research priorities may differ across organizations and projects. Academic investigators can make mechanistic explanation a primary scientific objective, drawing on institutional access to basic bench science, tissue repositories, and physiologic models. The well-documented case of a pneumonia-detection model that had partly learned to recognize hospital-specific image features correlated with disease prevalence serves as a cautionary reminder that statistical performance and clinical understanding can diverge quietly [37]. Exposing such hazardous divergences requires independent evaluators with a mandate and safeguards supporting independent scrutiny.

Second, AMCs are uniquely positioned to foster the interdisciplinary synthesis required to evaluate AI as a sociotechnical system [38]. Integrating expertise from medicine, computer science, ethics, law, and sociology is essential for assessing the real-world impact of AI on patient outcomes, clinical workflows, and health equity [39]. This holistic perspective is a core academic strength that can complement industry expertise in development and deployment. Evaluating AI as a sociotechnical system requires attention not only to model accuracy but also to workflow integration, liability implications, clinician cognitive load, and patient perception [40]. For example, a hypothetical AI triage tool that modestly improves diagnostic speed may simultaneously alter physician-patient communication patterns or shift responsibility boundaries in ways that are not captured by performance metrics alone. Academic institutions, with embedded departments of ethics, law, public health, and social science, are structurally equipped to examine these second-order effects. This interdisciplinary synthesis allows AMCs to assess not only whether an AI system works, but also how it reshapes clinical culture and institutional accountability [41].

Third, AMCs must strengthen their role as transparent evaluators of clinical AI [42]. Although many validation studies today are supported by industry, a legitimate source of concern regarding conflicts of interest, academic institutions remain essential for rigorous, methodologically independent assessment [43,44]. To strengthen methodological independence, we recommend explicit institutional firewalls, standardized reporting, and funding structures that reduce sponsor influence on study design, analysis, and publication. To improve transparency and comparability across studies, AMC-led evaluations should follow reporting guidelines appropriate to the study type: TRIPOD+AI (Transparent Reporting of a Multivariable Prediction Model for Individual Prognosis or Diagnosis—AI) for prediction-model studies [45], CONSORT-AI (Consolidated Standards of Reporting Trials–AI) for clinical trial reports [46] or SPIRIT-AI (Standard Protocol Items: Recommendations for Interventional Trials–AI) for clinical trial protocols [47]. Evaluation pipelines should include (1) external validation across settings and populations, (2) stress-testing for dataset shift and performance drift, and (3) explicit reporting of failure modes and subgroup performance because model behavior frequently changes across time, sites, and workflows [48]. Embedding these processes within institutional governance structures, similar in authority to institutional review boards (IRBs) or pharmacy and therapeutics committees, can help normalize AI oversight as a routine component of clinical safety rather than an ad hoc research activity [49]. Independence matters because otherwise evaluation becomes vulnerable to subtle distortions, including outcome measures chosen to flatter, comparator groups selected for contrast, and unfavorable subgroup performance left unpublished.

In practical terms, this may involve the establishment of dedicated Clinical AI Oversight Committees with multidisciplinary representation, including clinicians, data scientists, ethicists, legal scholars, and patient advocates [50]. Such bodies could evaluate procurement decisions, require standardized performance reporting, mandate periodic postdeployment audits, and ensure mechanisms for clinician override and incident reporting [51]. Importantly, governance should extend beyond initial approval to continuous monitoring, recognizing that model performance may drift as data distributions evolve [48]. Embedding AI oversight within institutional quality and safety infrastructures reframes algorithmic evaluation as an extension of patient safety rather than a purely technical exercise [52].

Finally, although academic medicine has its own history of ethical lapses, including research misconduct, conflicts of interest, and the exploitation of trainees, its nonprofit mission, public accountability, and responsibility for training future clinicians position AMCs as essential stewards of ethical AI oversight. This role does not assume moral superiority but recognizes that AMCs operate under public-facing obligations that differ structurally from those of commercial entities. Operationalizing this stewardship will require transparent governance frameworks, clearer conflict-of-interest policies, and institutional mechanisms that prioritize patient welfare over commercial incentives. This includes establishing standards for AI procurement, protecting patient privacy, ensuring human oversight in clinical decision-making, and addressing the algorithmic biases that can perpetuate health disparities.

## Re-Educating Clinicians for the Age of AI

To realize this vision, AMCs must adopt a concrete, 5-step action blueprint (Textbox 1). As illustrated in Textbox 1, this framework requires (1) longitudinal curriculum reform across UME, GME, and continuing medical education (CME); (2) standardized model evaluation pipelines for continuous clinical auditing; (3) precompetitive joint labs to develop shared benchmark datasets; (4) stackable microcredentials tied directly to clinical pathways; and (5) faculty upskilling combined with formal governance charters. These 5 steps reinforce one another, creating a longitudinal model of AI stewardship embedded within academic medicine. The framework also requires new partnership models that move beyond traditional sponsored research to create deeply integrated joint labs and data-sharing consortia, enabling academic researchers to work with industry-scale resources on precompetitive, public-interest challenges [30]. At the undergraduate level, AI literacy should emphasize 3 competencies: understanding model architecture at a conceptual level, recognizing common failure modes (including hallucination and bias amplification), and developing structured verification habits [53]. Assessment formats such as case-based critique of AI-generated clinical reasoning, simulated documentation review, and explicit “human-in-the-loop” reflection exercises can reinforce these competencies without transforming medical students into data scientists. Importantly, AI education should not be framed as technical supplementation but as an extension of professional formation [54]. In UME, this may include integrating AI literacy into preclinical foundational science courses and structured reflection on AI-assisted reasoning during clerkships. In GME, specialty-specific modules can address algorithmic triage, automated documentation, and human-override decision-making in supervised clinical environments. Faculty development programs should establish baseline AI governance competencies for attending physicians responsible for supervision and evaluation.

These 5 components address complementary implementation needs. Each is matched to a specific failure mode that recurs when academic centers approach clinical AI in an ad hoc manner. Curriculum reform addresses a workforce that cannot reliably appraise the tools it is given. Standardized evaluation pipelines address deployment of models that were never tested outside their development settings. Precompetitive joint labs address resource asymmetry that otherwise leaves academic evaluators without representative data. Microcredentials address the mismatch between multiyear curricular cycles and software that changes in months. Finally, comprehensive faculty upskilling paired with formal institutional governance charters addresses the absence of centralized institutional authority required to act on what evaluation reveals. These components are interdependent rather than strictly sequential. Governance authority and faculty development should begin early, while curriculum reform, evaluation pipelines, joint laboratories, and microcredentials mature iteratively. An AI-literate workforce supports rigorous clinical validation, and shared benchmark data can strengthen evaluation across institutions. Targeted microcredentials maintain workforce competencies as models and testing pipelines evolve. A formal governance charter connects these activities to a durable institutional safety function.

A practical way to prevent the blueprint from remaining aspirational is to define the minimum evidence and governance artifacts that a system must produce to earn routine clinical use. At a minimum, every deployed clinical AI tool should have a short “AI label” or model card adapted to clinical practice, stating intended use, population, training and validation cohorts, known failure modes, and required clinician oversight. That label should be paired with a local implementation dossier that specifies workflow integration points, override expectations, and a monitoring plan with explicit triggers for reassessment. These triggers can be simple: performance drift beyond a prespecified margin, sustained increases in override rates, documented safety events, or consistent subgroup disparities. Importantly, prespecified triggers linked to accountable action convert postdeployment monitoring from passive observation into an active safety process. This is also where AMCs can be distinctive without requiring computational scale. They can set a standard that procurement is conditional, not final: clinical AI tools are adopted provisionally, audited as they are used, and can be restricted or removed when monitoring reveals unacceptable performance. Over time, the institutional habit that matters most is not purchasing better tools, but making deimplementation possible.

Textbox 1. **A 5-step blueprint for responsible academic medical center (AMC) action in the era of generative AI.**

**1. Longitudinal curriculum reform**

- Integrate AI literacy and adversarial model evaluation throughout undergraduate medical education, graduate medical education, and continuing medical education, emphasizing clinical reasoning alongside AI outputs

**2. Standardized model evaluation pipelines**

- Implement institution-wide audit procedures for bias, reproducibility, external validity, and clinical safety. Publish results openly

**3. Precompetitive joint labs and shared benchmark datasets**

- Develop transparent partnerships where shared benchmarks and evaluation protocols (not proprietary products) are the outcomes

**4. Stackable microcredentials tied to clinical pathways**

- Issue AMC-based certifications such as “Clinical AI Safety,” “Responsible Large Language Model Use in Care,” and “AI-Augmented Clinical Workflows,” integrated into residency and fellowship training

**5. Faculty upskilling and governance charters**

- Create annual faculty certification in AI system interpretation and ethical oversight, and publish institutional AI governance charters defining decision-making authority and accountability

The barriers to implementing this model are predictable and should be named explicitly. First is resourcing. Sustained evaluation and monitoring require staff time, data access, and analytic capacity that many AMCs do not yet fund as a core safety function. Second is authority. Oversight groups that can only recommend but cannot condition procurement or require revalidation tend to become symbolic. Third is fragmentation. If evaluation sits in isolated departments rather than in quality and safety operations, results are harder to translate into institutional action. Fourth is faculty capability. Education reforms fail when supervisors are expected to assess competencies they do not themselves hold. These barriers point to realistic mitigation strategies. AMCs can start with a narrow portfolio of high-stakes systems, treat oversight as part of the patient safety infrastructure, and adopt a governance charter that defines procurement conditions, audit intervals, and escalation pathways. Faculty upskilling should be treated as a supervisory competence requirement, not as optional enrichment. Finally, AMCs can share the burden through consortia. Shared benchmark datasets, shared evaluation protocols, and pooled approaches to postdeployment monitoring reduce duplication and improve external validity without requiring each institution to build an assurance laboratory from scratch.

Progress can be made legible through a small number of measures: the proportion of supervising faculty who have completed formal AI safety training, the share of clinically deployed models that have passed an institutional evaluation, completion of an independent assessment before routine clinical deployment, followed by timely reassessment after material changes, and the existence of a chartered oversight body with documented authority over procurement and postdeployment audits. A major constraint is faculty capacity, since there are still few clinician-educators equipped to teach this material. Other barriers include the administrative load of standing up new committees, competition for protected curricular time, and the risk, already visible in early institutional experience, that an AI governance committee without a clearly bounded scope becomes overwhelmed and slow rather than authoritative [55].

Just as evidence-based medicine reshaped curricula in the late twentieth century, AI literacy must be embedded in clinical reasoning, ethics, and patient communication [56]. The objective is not automation dependency but reflective practice: clinicians who understand both the power and limitations of algorithmic systems and can articulate their use transparently to patients [57]. Generative AI represents a similar inflection point: clinicians must learn to interrogate algorithmic reasoning, recognize when outputs conflict with clinical context, and communicate uncertainty effectively to patients [58]. Embedding these competencies within professional identity formation can help ensure that AI becomes an instrument of augmentation rather than substitution [54,58]. The long-term legitimacy of AI-assisted care will depend, in part, on whether clinicians can articulate the rationale for AI-informed decisions and maintain patient trust in shared decision-making processes [59].

The proposed educational content can be made explicit across the training continuum. Drawing on the principles for AI in medical education issued by the Association of American Medical Colleges and on competency frameworks proposed in recent reviews and Delphi work [60-62], the relevant content can be organized into 4 domains that hold across the training continuum: foundational understanding of how models are built and where their outputs come from; practical and applied skills in using and supervising AI tools within real workflows; critical appraisal and verification, including recognition of failure modes such as hallucination, automation bias, and bias amplification; and ethical, legal, and communication competence, covering accountability, consent, equity, and how to convey algorithmic uncertainty to patients. These domains do not change from one stage of training to the next; what changes is their depth and the setting in which they are taught and assessed. Table 2 sets out the progression across UME, GME, and CME, pairing each stage with an assessment approach so that AI literacy is examined rather than merely delivered.

**Table 2.** A structured AI literacy competency outline across the medical education continuum.

Competency domain	Undergraduate medical education	Graduate medical education	Continuing medical education	Proposed assessment approach
Foundational understanding of AI systems	Conceptual model architecture; data provenance; what model training does and does not mean	Model types relevant to the specialty and the assumptions built into them	Briefings on new classes of models as they enter routine practice	Concept checks; brief structured written or oral items
Practical and applied use	Supervised exposure to AI-assisted reasoning during clerkships	Supervised use of triage, documentation, and decision-support tools	Procurement literacy and safe local adoption in one’s own setting	Observed use with documented override decisions
Critical appraisal and verification	Recognizing hallucination, automation bias, and bias amplification; structured verification habits	Appraising validation evidence; detecting performance drift in local data	Reading model cards and AI reporting standards (TRIPOD+AI^a^, CONSORT-AI^b^)	Case-based critique of AI-generated clinical reasoning
Ethics, equity, law, and communication	Accountability, consent, and equity; communicating algorithmic uncertainty to patients	Documentation standards; escalating suspected model error; awareness of liability	Institutional governance roles; participation in postdeployment audit	Reflection exercises; portfolio-based or audit-based assessment

a TRIPOD+AI: Transparent Reporting of a Multivariable Prediction Model for Individual Prognosis or Diagnosis—AI.

b CONSORT-AI: Consolidated Standards of Reporting Trials—AI.

Two design features of this structural architecture are worth explicit emphasis. First, competency-based assessment is treated as integral rather than optional. Any clinical competency that is taught but never examined tends not to survive contact with an already saturated curriculum. Consequently, each educational stage is tied to a proposed assessment approach: case-based critiques of AI-generated clinical reasoning, structured “human-in-the-loop” exercises in UME, supervised use with documented override decisions during GME, and formal audit-style or portfolio assessment in CME. Second, implementation is purposefully incremental and relies on existing structures to avoid the administrative resistance of creating standalone courses. AI literacy integrates into UME through existing foundational science modules and clerkship coursework rather than as a standalone course; GME can integrate algorithmic triage, automated documentation, and clinical override decision-making into specialty-specific teaching and into morbidity and mortality or quality-improvement conferences. Finally, CME can leverage modular, stackable microcredentials that practicing clinicians complete without leaving practice. Faculty development remains the critical rate-limiting step; AMCs must reframe AI governance proficiency as a baseline supervisory competence requirement for attending physicians rather than an optional faculty enrichment exercise, given that trainees cannot be validly assessed by supervisors who lack these core competencies themselves.

To effectively engage with an AI-enhanced world, faculty across disciplines must become digitally fluent, not only in using generative AI tools but also in understanding their architectures, limitations, and ethical implications [7]. In GME, specialty-specific integration becomes critical. Dermatology provides one example of a specialty in which AI image classifiers have demonstrated diagnostic potential [63]. Training must therefore include explicit discussion of when algorithmic outputs should be overridden, how to document AI-assisted decisions transparently, and how to escalate concerns about model error [52,58]. Embedding AI discussion within morbidity and mortality conferences or quality-improvement rounds may provide a natural institutional mechanism for cultivating a culture of oversight [64,65].

Academic institutions in both high-income countries and low- and middle-income countries (LMICs) face these pressures to embrace AI, though with distinct challenges. For LMICs, public-private partnerships and AI-focused centers of excellence may provide important pathways to meaningful participation in the global innovation ecosystem [7,66,67]. AI may support personalized learning in higher education [68]. By responding to learners’ prior knowledge, performance, and progress, AI tools may support tailored content, adaptive assessments, and timely feedback [69]. For CME, modular microcredentials in clinical AI safety, procurement literacy, and bias evaluation may offer a scalable pathway for practicing physicians to remain current. Such approaches acknowledge that AI systems will evolve more rapidly than traditional curricular cycles, requiring ongoing professional adaptation rather than one-time exposure during training [70]. Institutional metrics may help translate these educational reforms into measurable progress. Examples include tracking faculty completion of formal AI safety training, integrating AI oversight modules into residency accreditation reviews, and establishing cross-disciplinary committees to periodically reassess deployed systems. Without such structural reinforcement, AI education risks remaining aspirational rather than operational [71].

The shape of this work is not the same everywhere, and the difference between high-income and lower-income settings is worth making concrete. In high-income systems, access to tools is rarely the binding constraint. Dense electronic health records, an active regulatory apparatus, the evolving device pathway of the US Food and Drug Administration, the European Union AI Act, and a well-capitalized vendor market mean that the academic role is weighted toward validating and governing an already-crowded field of deployed products. Conversely, in many LMICs, the binding constraints sit earlier in the translational life cycle: highly fragmented, noninteroperable data systems, limited computational and connectivity infrastructure, and a shortage of trained clinician-informaticians. Policy and digital-health initiatives can support implementation, but pilot projects may face barriers to sustained integration into local health systems. Technically successful AI pilots targeting conditions such as tuberculosis, HIV, or maternal conditions may not be sustained after external funding ends without integration into local health systems and budgets [23]. For academic centers within these resource-constrained settings, institutional priorities must focus on capacity building, establishing regional centers of excellence, and anchoring regional, South-South, and North-South training partnerships to build local data science expertise, rather than on policing a saturated tool market. The 4 foundational academic roles are broadly applicable, with priorities and implementation determined locally.

## Conclusions

If AMCs fail to adapt, the trajectory of AI-enabled clinical practice will increasingly be shaped by proprietary systems whose underlying assumptions, biases, and optimization objectives remain largely invisible to clinicians. To prevent the erosion of public trust and ensure that algorithmic advances serve patient-centered goals, AMCs must actively assert their oversight role rather than withdraw from it. Over the next decade, the defining measure of academic medicine will not be its access to computational scale but its capacity to institutionalize responsible AI integration. By embedding rigorous validation pathways and prioritizing AI literacy for both faculty and trainees, institutions can shape national standards rather than reactively adapt to externally defined norms.

Ultimately, framing the future of medical AI as a competition between academia and industry is a false dichotomy. A more productive, complementary relationship is essential for responsible innovation (Table 3). This transformation mirrors the historical industrialization of pharmaceutical development: academic medicine did not attempt to compete with industry’s manufacturing capabilities; instead, it focused on understanding disease mechanisms, conducting independent clinical trials, and advocating for patient safety. By adopting a similar complementary posture today, AMCs can help shape the next era of health care, helping ensure that generative AI augments clinical reasoning while preserving the foundational human elements of medical practice.

**Table 3.** Complementary roles of industry and academia in medical AI innovation^a^.

Dimension	Industry focus	Academic medicine focus
Primary driver	Commercial ROI^b^ and scale	Scientific understanding and public health
Core strength	Rapid development and computational scale	Mechanistic inquiry, validation, and clinical integration
Innovation type	Pattern recognition and accuracy	Causal understanding, biological mechanisms, and safety
Data assets	Proprietary datasets and user feedback	Clinical cohorts, longitudinal outcomes, and diverse populations
Governance role	Regulatory compliance, risk management, and product liability	Patient safety standards, equity assessment, transparency, and independent evaluation
Success metrics	Accuracy, revenue, and adoption	Clinical utility, reproducibility, and fairness
Workforce role	Product training	Critical evaluation and clinical judgment
Public trust	Product reliability	Independent verification and bias detection
Shared responsibilities	Collaboration in joint laboratories, data-sharing partnerships, and regulatory engagement	Codevelopment of standards, shared evaluation pipelines, and partnership-driven oversight

a The table summarizes illustrative emphases rather than exclusive capabilities or responsibilities.

b ROI: return on investment.

## Abbreviations

AMC: academic medical center
CME: continuing medical education
CONSORT-AI: Consolidated Standards of Reporting Trials—AI
GME: graduate medical education
LMICs: low- and middle-income countries
NIST: National Institute of Standards and Technology
SPIRIT-AI: Standard Protocol Items: Recommendations for Interventional Trials—AI
TRIPOD+AI: Transparent Reporting of a Multivariable Prediction Model for Individual Prognosis or Diagnosis—AI
UME: undergraduate medical education
